# Medium term outcome of bipolar plasma vaporization in prostate cancer patients – A palliative modality of preserving spontaneous voiding

**Published:** 2012-12-25

**Authors:** B Geavlete, C Moldoveanu, G Niţă, F Stănescu, M Jecu, P Geavlete

**Affiliations:** "Sf. Ioan" Clinical Emergency Hospital, Department of Urology, Bucharest

**Keywords:** bipolar plasma vaporization, prostate cancer, complete urinary retention

## Abstract

**Objectives:**This retrospective analysis evaluated the efficiency, safety, and medium term postoperative results of bipolar plasma vaporization (BPV) in prostate cancer (PCa) cases associating complete urinary retention.

** Materials and Methods:** A series of 40 patients diagnosed with locally advanced or metastatic PCa and complete urinary retention requiring a Foley catheter indwelling underwent BPV aiming to restore spontaneous voiding. A total of 35 patients completed the one year evaluation protocol consisting of International Prostate Symptom Score (IPSS), quality of life score (QoL), maximum flow rate (Qmax) and post-voiding residual urinary volume (PVR), measured at 1, 3, 6 and 12 months after surgery.

** Results:** BPV was successfully performed in all cases with satisfactory efficiency, as confirmed by the mean operation time (42.8 minutes) and hemoglobin drop (0.7 g/dl). A fast and safe postoperative recovery period was described in this series (hematuria rate – 7.5%; mean catheterization period – 36 hours; mean hospital stay – 2.5 days; early-irritative symptoms’ rate – 15%). At 1, 3, 6 and 12 months, satisfactory values were determined in terms of IPSS, Qmax, QoL and PVR. These parameters emphasized a stable evolution throughout the entire follow-up, as 88.6% of the patients maintained spontaneous voiding.

** Conclusions:** The present trial confirmed the plasma-button vaporization as a promising therapeutic approach in PCa cases associating complete urinary retention. The technique displayed good efficacy, low perioperative morbidity, short convalescence, and satisfactory urodynamics and symptom score parameters during the one-year follow-up period.

**Abbreviations: **BPV – bipolar plasma vaporization; PCa – prostate cancer; TURP – transurethral resection of the prostate; BPH – benign prostatic hyperplasia; BOO – bladder outlet obstruction; LUTS – lower urinary tract symptoms; IPSS – International Prostate Symptom Score; QoL – quality of life score; Qmax – maximum flow rate; RV – post-voiding residual urinary volume

## Introduction

Prostate cancer (PCa) continues to pose a real problem for the contemporary medical care system. While preserving its place as an extremely important pathology in modern urology, PCa represents the most common solid neoplasm in male patients, as it was reported account for a proportion of 18.1% from the total number of diagnosed malignancies [**1**] and to currently constitute the second most common cause of cancer death in men [**2**]. 

 According to the EAU Guidelines, the monopolar transurethral resection of the prostate (TURP) represents the “gold-standard" endoscopic treatment in cases of bladder outlet obstruction (BOO) secondary to benign prostatic hyperplasia (BPH) [**3**]. The use of endoscopic resection in patients with PCa is rather remotely presented in literature, with very few available studies.

 On the other hand, PCa cases quite frequently describe severe lower urinary tract symptoms (LUTS) and urinary retention, thus affecting the quality of life of this category of patients and possibly resulting in a series of complications such as hydronephrosis, renal failure, and urinary tract infections [**4**]. From this point of view, the preservation of satisfactory voiding parameters by a minimally invasive approach can be considered as a rather important goal. As such, patients often present significant co-morbidities, the bipolar endoscopic electrosurgery, introduced while aiming to improve the performances of TURP and to reduce its complications, could be taken into consideration as a viable alternative.

The technique of bipolar plasma vaporization (BPV) was described both in literature [**5**] as well as in our own experience [**6**] as a viable therapeutic modality in cases of BOO related LUTS secondary to BPH. In the present study, we aimed to evaluate the efficiency, safety, and medium term postoperative results of this new procedure in PCa cases associating complete urinary retention. Following the introduction of this new approach in an initial series [7-11], the clinical results were analyzed among a larger number of patients and during a prolonged follow-up period, as the one year outcome of this endoscopic alternative was considered particularly relevant.

## Materials and Methods

A retrospective analysis was performed aiming to assess a series of 40 locally advanced or metastatic PCa patients. The mean patients’ age was 72.6 years (range 56-81).

 A standard investigation protocol which included general clinical evaluation with digital rectal examination (DRE), blood tests, prostate specific antigen (PSA), urinalysis, urine culture as well as abdominal and transrectal ultrasound was applied in all cases.

 The Olympus SurgMaster UES-40 bipolar generator, saline continuous flow irrigation, the OES-Pro bipolar resectoscope and the “button" type vaporesection electrode (Olympus Europe, Hamburg, Germany) were used in all cases. 

 The inclusion criteria consisted of complete urinary retention requiring a Foley catheter indwelling and previously diagnosed as locally advanced (stage T3-T4) or metastatic (N+ or/and M+) prostate cancer. Only T3 patients without indication for radical treatment were included in this series. Hormonal and/or radiotherapy were applied in all cases for at least 3 months before the procedure and the decision for surgery depended on patient’s choice. All the interventions were carried out under spinal anesthesia.

**Fig. 1:**
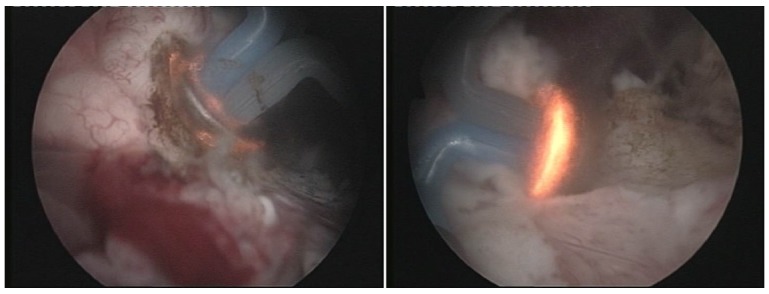
Plasma vaporization of the prostatic tissue

**Fig. 2:**
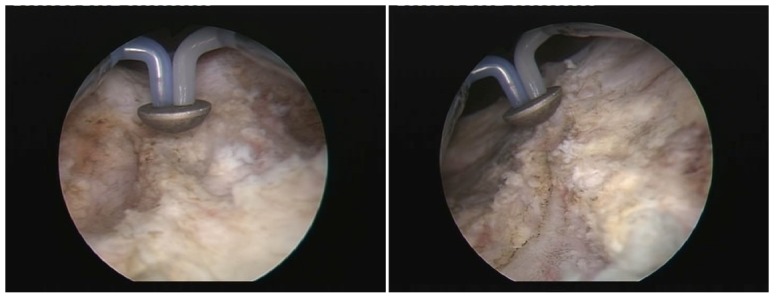
Specific endoscopic image of the invaded prostatic capsule after bipolar vaporization

**Fig. 3:**
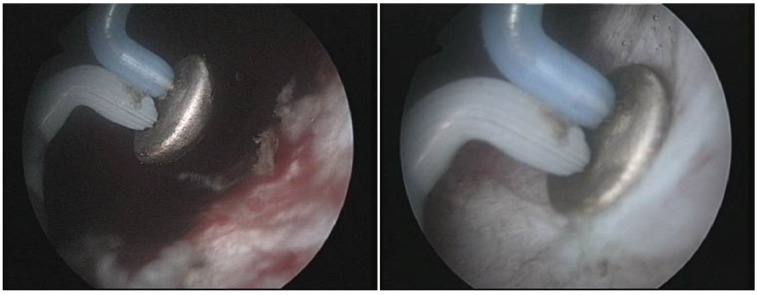
Plasma-button coagulation of the remaining bleeding sources

 During BPV, the hemispherical shape electrode displaying a plasma corona on its surface was gradually moved in close contact with the prostatic tissue (**Fig. 1**), which was vaporized layer by layer until reaching the prostatic capsule (the so-called “hovering" technique) (**Fig. 2**). A virtually blood-less vaporization was produced at 280-320 W and larger vessels’ hemostasis was achieved at 120-140 W (**Fig. 3**). In all cases, a 20F Foley catheter was placed at the end of the procedure.

All patients were assessed at 1, 3, 6 and 12 months after surgery by International Prostate Symptom Score (IPSS), quality of life score (QoL), uroflowmetry (evaluating the maximum flow rate – Qmax) and abdominal ultrasound (measuring the post-voiding residual urinary volume – PVR). During the entire follow-up period, hormonal therapy was continued in all cases in which it was previously applied as well as in patients with inadequate response to radiotherapy. 

## Results

 The mean preoperative prostate volume measured by transrectal ultrasound was of 54.2 grams. BPV was successfully performed in all cases within a mean operation time of 42.8 minutes. The mean hemoglobin drop described during surgery was of 0.7 g/dl, while the rates of intraoperative bleeding (5%) and capsular perforation (5%) remained low. 

 The safety profile of the procedure remained satisfactory during the immediate postoperative period, as shown by the reduced hematuria rate (controlled by conservative measurements – 7.5%), absence of blood transfusion necessity and fast recovery (mean catheterization period – 36 hours; mean hospital stay – 2.5 days). 

 There were no cases of clot retention or re-hospitalization for secondary hemorrhage. A total of 7.5% of the patients required re-catheterization due to postoperative acute urinary retention and subsequently regained spontaneous voiding upon catheter removal one week after discharge. Additionally, the rate of early irritative symptoms (15%) and urinary tract infections (5%) remained within the accepted limits of transurethral surgery, while no cases of urinary incontinence were described.

 A number of 35 patients completed the one year’ evaluation protocol. One patient died from cancer, another from associated co-morbidities and three cases were lost from the follow-up. In total, 88.6% of the patients maintained spontaneous voiding during the entire 12 months of the study period. Re-catheterization was at some point required in two cases, re-intervention for haemostatic purposes was necessary in one patient and for removing bladder outlet obstruction in another case. 

 At the 1, 3, 6 and 12 months check-ups, satisfactory values were determined in terms of IPSS, Qmax, QoL and PVR and these parameters emphasized a stable evolution throughout the follow-up evaluation period (**Table 1**). 

**Table 1:** Follow-up parameters

Parameters		Follow		
	1 month	3 months	6 months	12 months
IPSS	6.2	5.7	5.4	5.2
QoL	1.6	1.4	1.4	1.5
Qmax	21.3 mL/s	22.1 mL/s	22.3 mL/s	21.9 mL/s
PVR	37 mL	30 mL	33 mL	28 mL

## Discussion

The bipolar electro-surgery in general and transurethral resection in saline (TURis) in particular appeared to be equivalent to the monopolar technology in terms of surgical efficacy in BPH and bladder tumors’ cases, with remarkable safety advantages and no increase in the incidence of urethral strictures [**8**]. 

 In this regard, the bipolar resection was described to offer functional results comparable to standard TURP as well as the advantages of improved hemostasis, TUR syndrome prevention and reduced overall complications [**9**]. As far as PCa is concerned, Sevriukov et al. successfully applied TURis in patients with diagnosed prostatic malignancy, described good functional results, and reduced perioperative morbidity, thus suggesting a possibly viable application of bipolar electrosurgery in this particular category of cases [**10**]. 

 Unfortunately, not all literature data confirmed the superiority of bipolar electrosurgery over the conventional TURP in PCa patients. For example, a trial by Michielsen et al. showed that, despite the promising experimental results of superior haemostatic abilities and increased coagulation depth, bipolar technology failed to reduce the amount of blood loss when compared to monopolar resection in a cohort of patients undergoing oral anticoagulation therapy. As of such, similar blood transfusions requirements, mean number of transfused units and clot retention rates were described for TURis and TURP [**11**].

 However, older studies underlined the fact that, in patients with acute urinary retention secondary to prostate carcinoma in whom hormonal manipulation was thought appropriate due to the local tumor bulk or metastatic disease, channel TURP may induce additional morbidity. Consequently, some authors stated that limited palliative resection should be held in reserve for those patients unable to void after hormonal manipulation [**12**].

 On the other hand, radical transurethral resection of the prostate was at one point introduced as an alternative therapy for the PCa treatment. Basically, some studies showed that prostate cancer can be endoscopically ablated, thus providing reasonable oncological results. The outcome with respect to survival and PSA recurrence was described as compared with the results of other treatment options [**13**]. An extended clinical analysis of this perspective established that prostatic malignancy could be resected transurethrally in a radical manner, relatively comparable to open surgery, while obtaining a significantly reduced urinary incontinence rate [**14**].

 Further along this line of endoscopic management, a satisfactory cancer control was reported in the available literature subsequent to focal TUR performed in localized prostate cancer patients. The respective type of approach was underlined as a potential option of PCa focal therapy with minimum adverse effects on urinary continence and erectile function [**15**].

 From our point of view, based on the previously determined satisfactory results of the plasma vaporization in cases of BPH [**16**] and large bladder tumors [**17**], this technique was evaluated in a series of locally advanced or metastatic PCa patients suffering from complete urinary retention as a potentially effective modality of removing BOO [**7**].

**Fig. 4:**
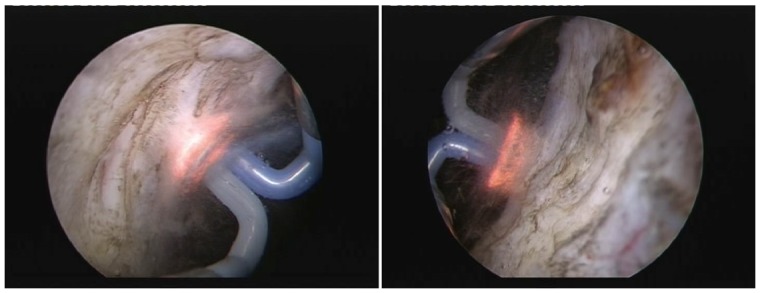
Vaporization of the remaining tissue close to the prostatic capsule

 Subjectively speaking, in our experience, the bipolar vaporization offered the advantages of remarkable intraoperative visibility due to reduced bleeding. During the procedure, a detailed visual differentiation of the normal prostatic and malignant tissue structures as well as of the capsular fibers subsequent to the plasma vaporization process was constantly obtained (**Fig. 4**).

**Fig. 5:**
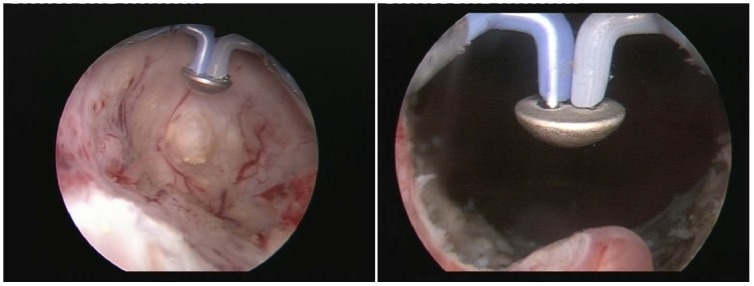
Pre- and postoperative aspect of the prostatic fossa

The final postoperative aspect revealed a large prostatic fossa and a particularly smooth surface and sharp margins of the vaporization area, without irregularities or obstruction (**Fig. 5**). 

Consequently, we determined reduced capsular perforation (5%) and intraoperative bleeding (5%) rates for this technique [**7**].

Concerning the literature data assessing the endoscopic approach in PCa patients, according to a study by Reuter et al. on 1017 cases that underwent standard TUR, the blood transfusion rate was of 4.4% and the rate of endoscopic revision for secondary bleeding was of 2.5% [**14**]. Since in our study, however based on a limited number of patients, no such complications occurred, BPV seemed to emphasize an apparently superior safety profile in PCa related BOO by comparison to the monopolar resection [**7**]. 

Also, the mean catheterization period and hospital stay determined in the present series (36 hours and 2.5 days) successfully matched our previous experience in BPH treatment [**6**] as well as the published data referring to bipolar and respectively monopolar TUR applied in average BPH patients (60.5 hours and 3.02 days; 81.8 hours and 3.88 days [**18**]) as well as in prostate cancer patients (2.0 and 5.8 days versus 1.6 and 4.6 days, respectively [**19**]). 

The surgical time specific for the plasma-button vaporization approach (42.8 minutes) followed the pattern of PCa related monopolar TUR (40.0 minutes) and emphasized a relative improvement when related to bipolar resection (49.1 minutes) at somewhat similar preoperative prostate volumes (54.2 versus 51.0 and 58.6 grams, respectively [**19**]).

On the other hand, the BPV related mean hemoglobin drop (0.7 g/dL) was somewhat lower than the one reported for either monopolar (1.2 g/dL) or bipolar (1.3 g/dL) TUR [**19**]. Additionally, a satisfactory percentage of postoperative urinary retention was determined of the plasma-button approach (7.5%) when compared to both monopolar (15%) and bipolar (27%) resection [**19**]. Moreover, the fact that no patient in the present series required a long term indwelling catheter could be considered important, as this category of cases has been found with a frequency of up to 23% [**19**]. 

Most importantly, the postoperative results at 6 months regarding the IPSS and Qmax obtained among our study group (5.4 and 22.3 ml/s) can be considered satisfactory and comparable to the literature data evaluating monopolar TURP and TURis in BPH cases (5.8 and 21.9 ml/s, 5.5 and 22.5 ml/s, respectively [**20**]). 

Further along this perspective, the available studies showed that palliative TURP can be safely performed in advanced prostate cancer patients, thus obtaining a significant improvement in urinary symptoms [**21**]. In this regard, according to a trial by Engelhardt et al., an IPSS decrease from 21.1 to 11 was determined after a follow-up period of 14 months [**21**] while our study confirmed an IPSS of 5.2 at the 12 months’ evaluation. 

Also, the fact that BPV provided stable spontaneous voiding during the entire follow-up in all patients with good urodynamics and symptom score’ parameters can be seen as an encouraging argument in favour of this new technique. Of course, longer follow-up periods and more extensive trials will be required in order to establish the long-term advantages and general viability of the method as a palliative therapeutic approach in PCa. 

## Conclusions

It could be concluded that, in light of the present findings, the plasma-button vaporization technique appears to represent a promising therapeutic approach in PCa patients with complete urinary retention.

The procedure displayed good efficacy, low perioperative morbidity, short convalescence, stable clinical evolution and satisfactory urodynamics and symptom score parameters during the one-year follow-up period.

The remarkably efficient prostatic tissue vaporization, excellent visibility, reduced intra- and postoperative bleeding, brief catheterization period and hospital stay, acceptable frequency of complications and superior medium term results might constitute reliable arguments in favour of this new type of intervention.

 The elevated percentage of patients benefitting from spontaneous voiding during the 12 months following BPV sustain its’ viability as an effective means of improving the quality of life in patients with locally advanced or metastatic prostate cancer.
